# Phylogeography of human Y-chromosome haplogroup Q3-L275 from an academic/citizen science collaboration

**DOI:** 10.1186/s12862-016-0870-2

**Published:** 2017-02-07

**Authors:** Oleg Balanovsky, Vladimir Gurianov, Valery Zaporozhchenko, Olga Balaganskaya, Vadim Urasin, Maxat Zhabagin, Viola Grugni, Rebekah Canada, Nadia Al-Zahery, Alessandro Raveane, Shao-Qing Wen, Shi Yan, Xianpin Wang, Pierre Zalloua, Abdullah Marafi, Sergey Koshel, Ornella Semino, Chris Tyler-Smith, Elena Balanovska

**Affiliations:** 1 Vavilov Institute of General Genetics, Moscow, Russia; 2 Research Centre for Medical Genetics, Moscow, Russia; 3 YFull service, Moscow, Russia; 4 National Laboratory Astana, Nazarbayev University, Astana, Republic of Kazakhstan; 5 Department of Biology and Biotechnology “L. Spallanzani”, University of Pavia, Pavia, Italy; 6 Gene by Gene, Ltd, Houston, USA; 7 Ministry of Education Key Laboratory of Contemporary Anthropology, School of Life Sciences, Fudan University, Shanghai, China; 8 Department of Criminal Investigation, Xuanwei Public Security Bureau, Xuanwei, China; 9 Lebanese American University, Beirut, Lebanon; 10 Full Genomes Corporation, Rockville, MD USA; 11 Faculty of Geography, Lomonosov Moscow State University, Moscow, Russia; 12 The Wellcome Trust Sanger Institute, Wellcome Genome Campus, Hinxton, UK

**Keywords:** Y-chromosome, Haplogroup Q, Population genetics, Genetic genealogy, Ashkenazi, Phylogeography, Gene geography

## Abstract

**Background:**

The Y-chromosome haplogroup Q has three major branches: Q1, Q2, and Q3. Q1 is found in both Asia and the Americas where it accounts for about 90% of indigenous Native American Y-chromosomes; Q2 is found in North and Central Asia; but little is known about the third branch, Q3, also named Q1b-L275. Here, we combined the efforts of population geneticists and genetic genealogists to use the potential of full Y-chromosome sequencing for reconstructing haplogroup Q3 phylogeography and suggest possible linkages to events in population history.

**Results:**

We analyzed 47 fully sequenced Y-chromosomes and reconstructed the haplogroup Q3 phylogenetic tree in detail. Haplogroup Q3-L275, derived from the oldest known split within Eurasian/American haplogroup Q, most likely occurred in West or Central Asia in the Upper Paleolithic period. During the Mesolithic and Neolithic epochs, Q3 remained a minor component of the West Asian Y-chromosome pool and gave rise to five branches (Q3a to Q3e), which spread across West, Central and parts of South Asia. Around 3–4 millennia ago (Bronze Age), the Q3a branch underwent a rapid expansion, splitting into seven branches, some of which entered Europe. One of these branches, Q3a1, was acquired by a population ancestral to Ashkenazi Jews and grew within this population during the 1st millennium AD, reaching up to 5% in present day Ashkenazi.

**Conclusions:**

This study dataset was generated by a massive Y-chromosome genotyping effort in the genetic genealogy community, and phylogeographic patterns were revealed by a collaboration of population geneticists and genetic genealogists. This positive experience of collaboration between academic and citizen science provides a model for further joint projects. Merging data and skills of academic and citizen science promises to combine, respectively, quality and quantity, generalization and specialization, and achieve a well-balanced and careful interpretation of the paternal-side history of human populations.

**Electronic supplementary material:**

The online version of this article (doi:10.1186/s12862-016-0870-2) contains supplementary material, which is available to authorized users.

## Background

The markers of the male-specific region of the Y-chromosome (MSY) exhibit the highest inter-population diversity in the human genome. Though some other genome regions contain more polymorphic sites, Y-chromosomal markers exhibit higher variation of allele frequencies across different populations than markers on other chromosomes; this phenomenon is generally attributed to strong genetic drift in combination with the patrilocality common to most human populations; indeed, in matrilocal populations the pattern is different [1–3]. Thus for most human populations, Y-chromosomal markers form one of the most informative tools for tracing their demographic history [4–6]. Through numerous academic publications, the study of Y-chromosome variation has grown into an important field of population genetics. The MSY is also a valuable tool for tracing individual genealogies, and it is thus widely used in genetic genealogy, one of the most popular fields of citizen science. Individual and population origin are interrelated, resulting in mutual interactions: for example, academic papers in the field of population genetics are widely discussed on genealogical internet forums [7, 8], and, similarly, the Y-chromosome tree of the International Society of Genetic Genealogy [9] has become a standard resource for population geneticists [10–15].

“Citizen science” in the Oxford English Dictionary is defined as: “scientific work undertaken by members of the general public, often in collaboration with or under the direction of professional scientists and scientific institutions” [16, 17]. Genetic genealogy is one of the manifestations of citizens’ activity in the field of scientific research [18]. However, direct collaboration between population geneticists and genetic genealogists has been limited [19–21].

The information value of the MSY often depends on discovering SNPs which subdivide haplogroups with broad geographic distribution into branches, revealing the fine population structure. Progress in such discoveries has recently moved from a slow linear phase to a rapid exponential phase due to the use of next-generation sequencing technologies, resulting in a number of high-quality papers applying this approach to overall patterns of paternal population history [10, 11, 15, 22–25] and to the phylogeography of specific haplogroups [12, 26–28].

To the best of our knowledge, only a few studies based on full Y-chromosome sequencing include Y-chromosomes belonging to haplogroup Q [24, 29], and none of them focused specifically on it. This haplogroup is well known because it makes up about 90% of indigenous Native American Y-chromosomes, but is also present in North Eurasia [6]. A rare clade called Q3 has been detected in Y-chromosomal resequencing studies [24, 29] and was found at low frequencies in Iraqis [30], Iranians [31], Israelis [32, 33], Indian Brahmins [34] and Pashtuns [35]. Rare haplogroups can be very informative for tracing human migration routes [36–38], and here we concentrate on improving resolution in the Q3-L275 portion of the first Q1’2 *versus* Q3 bifurcation of haplogroup Q-M242 [9, 39]. We combined the efforts of population geneticists and genetic genealogists to use the potential of full Y-chromosome sequencing for reconstructing haplogroup Q3-L275 phylogeography and suggest possible linkages to events in population history.

## Results

**Fig. 1:**
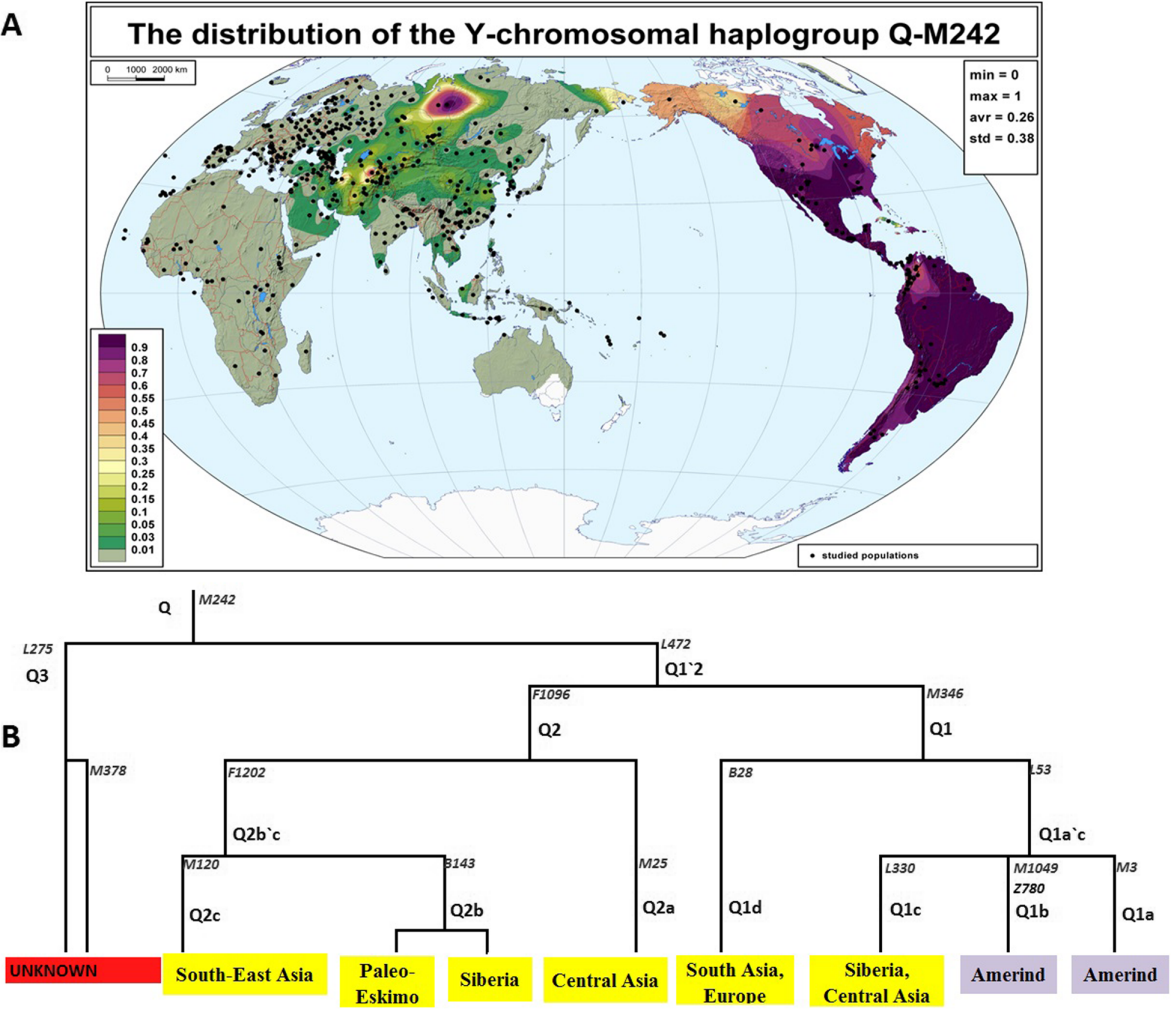
Overview of haplogroup Q. **a** Global frequency distribution map of haplogroup Q-M242. The map was constructed using Q-M242 frequencies in 480 populations (total sample size 65,528). **b** Phylogenetic structure of haplogroup Q-M242 (modified from [24])

We constructed a detailed frequency distribution map of haplogroup Q-M242 (Fig. 1a) which demonstrated, in agreement with the previous studies, the haplogroup’s presence throughout Asia and the Americas with frequency peaks in America and Central Siberia. There are three major trunks: Q1, Q2, and Q3 (Fig. 1b). The first two trunks split into multiple branches, some of which are known to be purely Asian, while others are both Asian and (extant or extinct) American (Fig. 1b and references therein). Little is known about the distribution of the third branch known formerly as Q1b and now as Q3 [24, 40, 41], which is in focus of this study.

## Distribution of Q3-L275

**Fig. 2:**
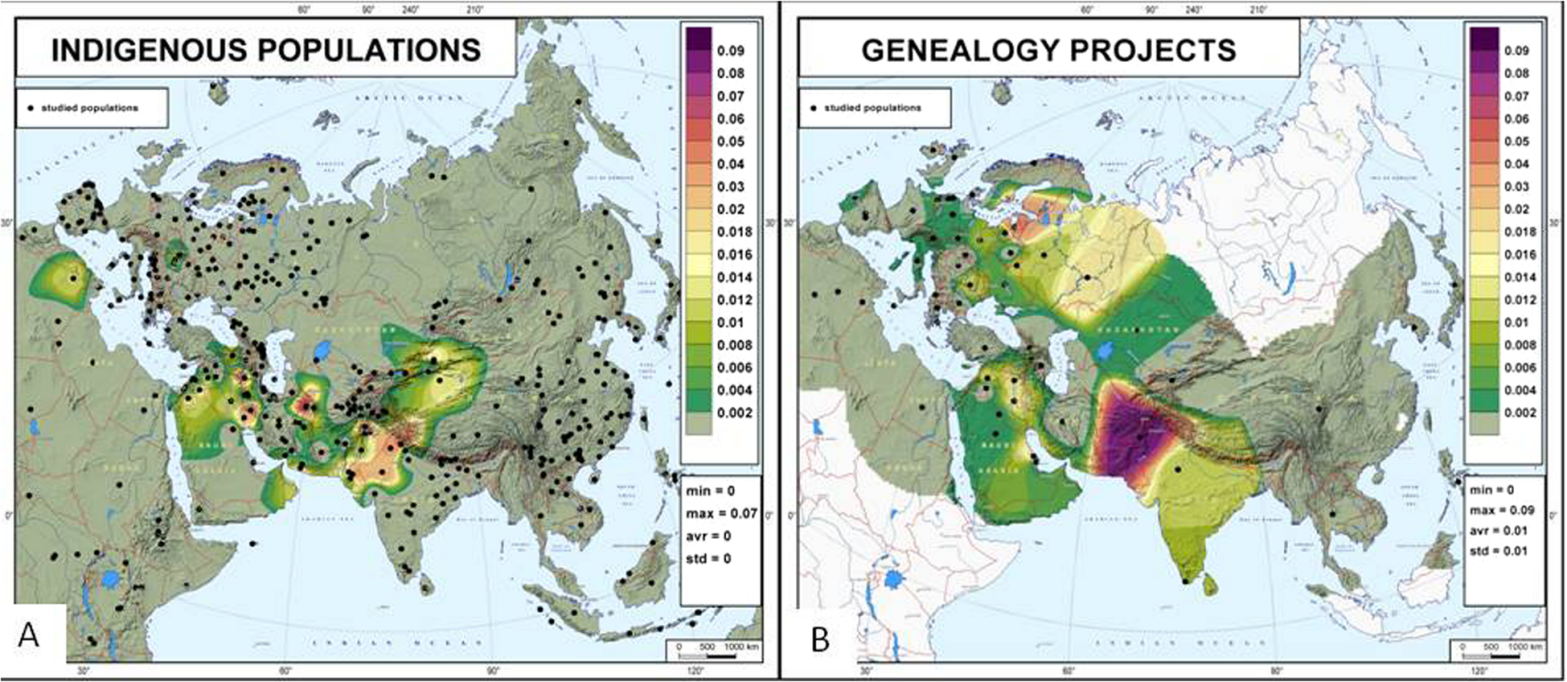
Frequency distribution map of haplogroup Q3-L275. **a** Data from indigenous populations from academic papers (total sample size 11,566). **b** Data from genealogical projects (Jewish projects not included, total sample size 23,730). The plotted frequencies were calculated as number of Q3-L275 carriers in the corresponding genealogical project divided to the total number of persons in the project

To evaluate the Q3 distribution, we analyzed both academic data from indigenous populations and data from genealogical projects. The frequency distribution map based on academic data (Fig. 2a) reveals that haplogroup Q3-L275 is confined to West Asia and neighboring parts of Central and South Asia – mainly Pakistan, West India, and up to 7% in Iran (see also Table 1). The map based on genealogical project data (Fig. 2b) also reveals the presence of haplogroup Q3-L275 in West Asia and neighboring areas, with a maximum frequency in Pakistan, but also throughout Europe. When data on Ashkenazi Jew genealogical projects are included (see Methods for details), the Q3 frequencies in Europe become almost as high as in West Asia (Additional file 1: Figure S1).

**Table 1:** Frequencies of haplogroup Q3-L275 in indigenous populations and population-based genealogical projects

Indigenous populations				Genealogical projects		
Population/Country	Frequency (%)	*N*	Reference	Genealogical project	Frequency (%)	*N*
Europe						
Austria-Hungary	1,3	76	[32]	Alpine DNA Project	0,3	384
Slovak	0,6^a^	473	[67]	Anglo-Saxon	0,2	415
				Benelux	0,4	480
Czech	0	120	[68, 69]	Czech DNA	0,3	381
French	0	130	[70–73]	French Heritage franiais	0,1	2613
German	0	499	[68, 69, 72, 74]	German DNA Project	0,2	2840
Iberian	0	798	[75]	Iberian Peninsula DNA Project	0,1	1829
Italian	0	55	[72, 76]	Italy	0,4	825
Latvian	0	86	[71]	Latvia DNA	4,6	175
Lithuanian	0	339	[77, 78]	Lituania Propria	0,4	504
				Nethelands Dual DNA Project	0,2	405
Polish	0	964	[68, 70, 71, 74]	Polish Family Tree DNA	0,9	3074
Portuguese	0	30	[79]	Portugal DNA Project	0,3	372
Romanian	0	67	[80]	Romania	1,2	167
Russian	0	2119	[71, 81–83]	Russia DNA	1,1	1309
				Russia-Slavic DNA Project	1,2	432
Tatar	0	56	[81, 84]	Tatarstan	1,7	180
Ukrainian	0	911	[70, 78]	Ukrainian DNA Project	0,5	434
Jewish						
Ashkenazi	5,1	441	[85]	Ashkenazi Iberian	1,5	758
Cohanim	0,9	215	[32]	Jewish DNA	2,5	1796
Israelites	3,7	738	[32]	Jewish German	5,7	244
				Jewish Ukraine West	2,3	1006
				Jewish Prague	2,5	80
				Gesher Galicia - Jewish DNA	3,6	308
West Asia						
Arab	2,1	143	[30]	Arabian-Gulf	0,2	1233
Assyrian, Iran	2,6	39	[86]	Assyrian	1,3	76
Azeri, Iran	1,6	63	[86]	Azerbaijan DNA Project	0	80
Bandari, Iran	0,8	131	[31]	Armenian DNA Project	0,6	634
Gheshmi, Iran	2,0	49	[31]	Aramaic DNA Project	1,2	85
Iranian, East	0,8^a^	124	[31]	Bahrain	1,9	54
Iranian, West	1,5^a^	200	[31]	Iranian DNA Project	0	134
Iraqi	1,9	154	[30]	Iraq DNA Project	1,3	524
Jordanian	1,5^a^	275	[67]			
Lebanese	1,2^a^	145	[67]			
Lebanon	0,3^a^	334	[86]	Lebanon-Syria-Palestine-Jordan	0	323
Lur, Iran	3,9	50	[31]			
Persian, Iran	7,1	70	[31]	Middle East DNA Project	0,3	1950
Syrian	1,5^a^	65	[67]			
Turkish	1,0^a^	585	[67]	Turkey	0	319
Central Asia						
Hazara	1,0^a^	101	[87]			
Hazara, Xingjiang	0	53	[88]			
Kazakh, Xingjiang	0	72	[89]	Kazahstan DNA-project	0,3	606
Kyrghyz	0	13	[90]	Kirgiz DNA-project	0	72
Pashtun	0	87	[87]			
Uyghur, Xinjiang	4^a^	194	[89]			
Uyghur, Xinjiang	2,7^a^	89	[88]			
South Asia						
Afghanistan	0,7	190	[35]	Afghan-Pakistani DNA Project	8,7	103
Afghanistan, South	0,5	146	[35]			
Himachal Pradesh	5^a^	59	[34]			
India, Central	0^a^	72	[91]	Syrian Christians of Kerala,India	1,0	104
India, East	0^a^	128	[91]			
India, North	0^a^	80	[91]			
India, North Rajasthan	2,3^a^	44	[34]	India subcontinent DNA Project	1,1	470
India, South	0^a^	303	[91]			
Pakistan	1,1^a^	177	[91]			
Pakistan, North	1,2^a^	86	[91]			
Pakistan, South	1,1^a^	91	[91]			
Pathan, Pakistan	2,6^a^	270	[92]			
Punjab	3,5^a^	57	[34]			
Sindhi	4,8^a^	21	[87]			
North Africa						
Morocco	1,2^a^	83	[32]	Egypt	0	144

The table contains populations where haplogroup Q3-L275 was found (zero frequencies are shown only for populations where haplogroup Q3 was revealed in the alternative dataset)

^a^These samples were assigned to haplogroup Q3-L275 based on their STR haplotypes

## Phylogenetics of Q3-L275

**Fig. 3:**
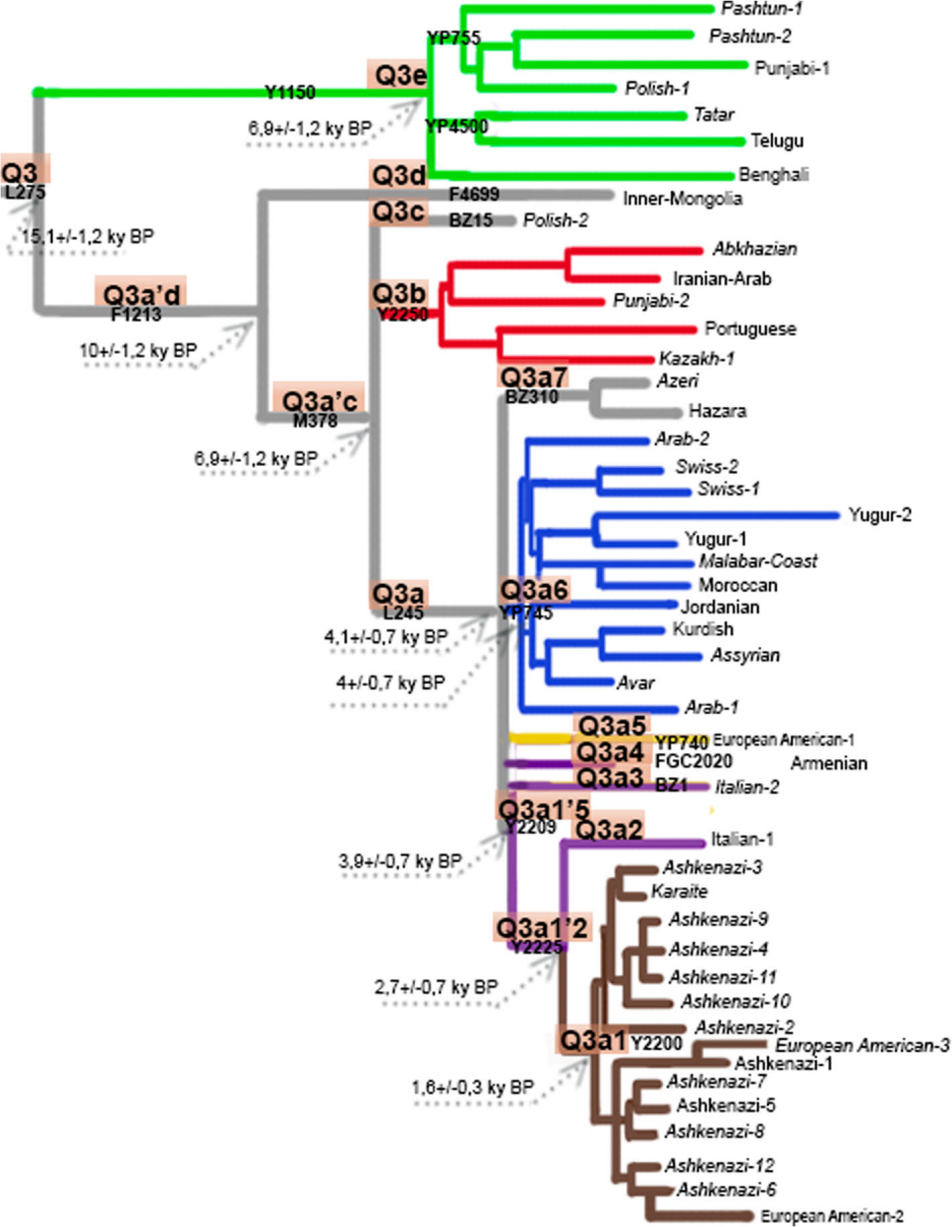
Phylogenetic tree of haplogroup Q3-L275 based on full sequences of the Y-chromosome. Additional file 2: Table S1 provides the detailed version of this tree. Notes contain ages (in kilo years) of the principal clades. Samples used for age calculations are shown in italic

We sequenced a large portion of the Y-chromosome in 47 samples belonging to haplogroup Q3 and in one outgroup Q1 sample. This dataset allowed us to reconstruct a detailed phylogenetic tree of haplogroup Q3, calibrated using a mutation rate of 0.78 × 10^−9^ mutations per bp per year [26] (Fig. 3). The first split in the tree occurred in the Upper Paleolithic, around 15,000 years ago (ky BP), giving rise to the Q3e branch and its brother Q3a’d branch (Table 2, Fig. 3). The first split in the latter generated Q3d and the remaining Q3a’c around 7 ky BP; Q3a’c subsequently split into Q3a, Q3b, and Q3c. Thus, at the beginning of the Neolithic period, the five main branches within Q3 already existed. Q3c and Q3d were represented in our dataset by single sample each; Q3e split into sub-branches around 6-7 ky BP; Q3b split around 5 ky BP; Q3a did not exhibit splits till 3-4 ky BP when it demonstrated a remarkable set of phylogenetic splits (Table 2).

**Table 2:** Names and ages of haplogroup Q3 clades

This study	ISOGG	YFull	Defining marker	Age
Q3	Q1b	Q1b	L275	15100 ± 1200
Q3e	Q1b2	Q-Y1150	Y1150	6900 ± 1200
Q3d	-	Q-F4699	F4699	^a^
Q3a'c	Q1b1	Q-M378	M378	6900 ± 1200
Q3c	-	Q-BZ15	BZ15	^a^
Q3b	Q1b1b	Q-Y2250	Y2250	5100 ± 1100
Q3a	Q1b1a1	Q-L245	L245	3700 ± 900
Q3a7	-	Q-BZ310	BZ310	^a^
Q3a6	Q1b1a1a2	Q-YP745	YP745	4000 ± 700
Q3a1'5	Q1b1a1a1	Q-Y2209	Y2209	3000 ± 700
Q3a3	-	Q-BZ1	BZ1	^a^
Q3a4	-	Q-FGC2020	FGC2020	^a^
Q3a5	Q1b1a1a1b	Q-YP740	YP740	^a^
Q3a1’2	Q1b1a1a1a	Q-Y2225	Y2225	^a^
Q3a1	Q1b1a1a1a1	Q-Y2200	Y2200	1600 ± 300
	Q1b1a1a1a1a	Q-Y2197	Y2197	1170 ± 170
	Q1b1a1a1a1b	Q-YP1003	YP1003	1500 ± 400

^a^these ages were not calculated because corresponding samples were not sequenced with the BigY technology (see Methods)

These events started with the ternary split of Q3a into Q3a1’5, Q3a6, and Q3a7 (4.1+/- 0.7 ky BP). Two of these branches themselves split immediately: the quarternary split of Q3a1’5 (into Q3a1’2, Q3a3, Q3a4, and Q3a5) occurred 3.9+/-0.7 ky BP and the ternary split of Q3a6 occurred 4.0+/-0.7 ky BP. Thus, from 7,000 to 4,000 years ago there were no splits within the Q3a lineages examined, but around 3,000–4,000 years ago this branch has split into seven sub-branches. Though phylogenetic analysis identified the sequence of these splits, the confidence intervals of their dates overlap considerably, showing that these events occurred within a narrow time interval.

The tree demonstrates a final set of events within the last two thousand years: Q3a1’2 split into Q3a1 and Q3a2; Q3a1 split into two sub-branches 1,6+/-0,3 ky BP, and these sub-branches in turn split between the 2nd and 10th centuries AD (Table 2, Additional file 2: Table S1).

## Phylogeography of Q3-L275

**Fig. 4:**
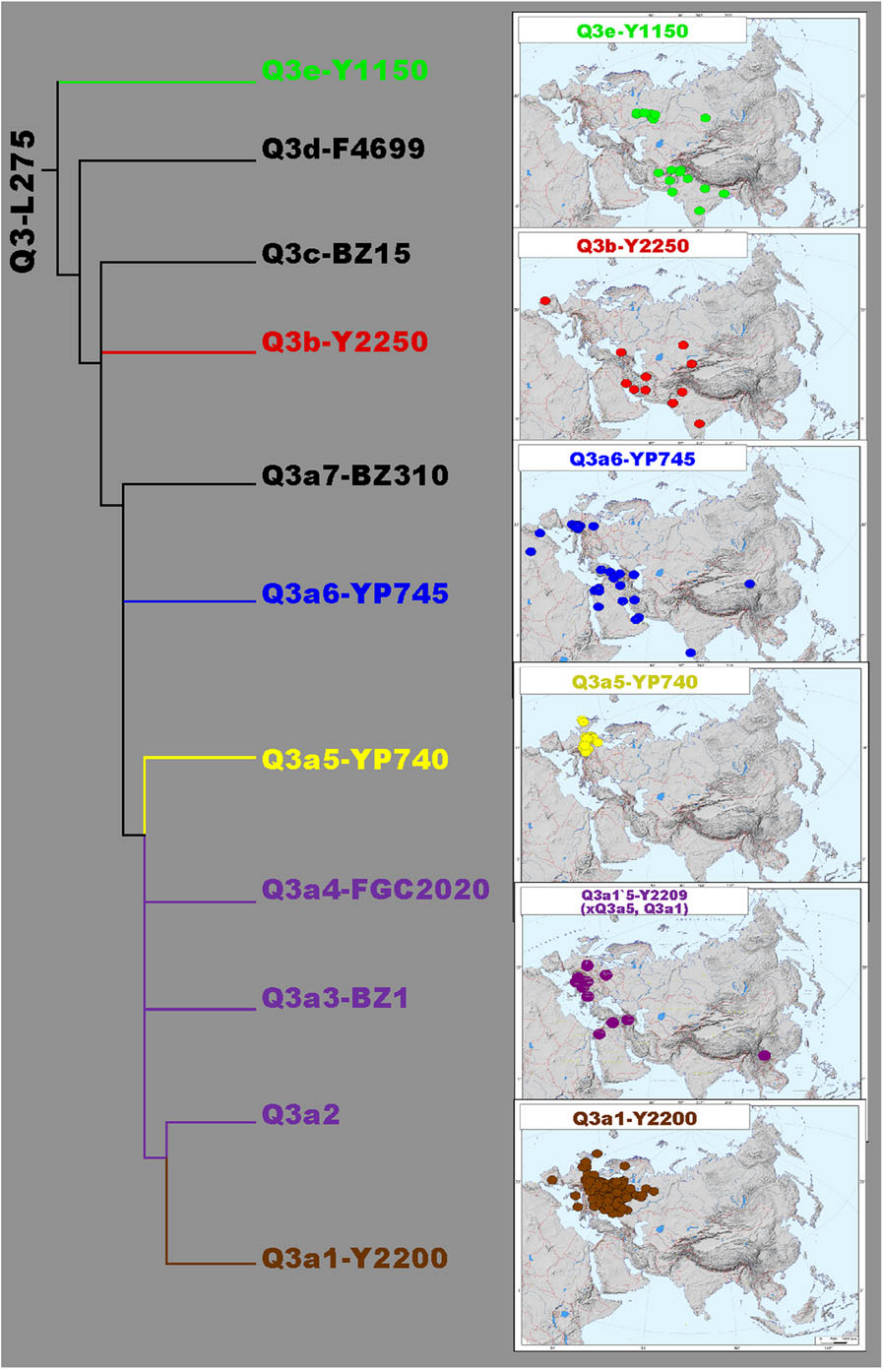
Phylogeographic summary: geographic locations of individual branches. Colors of branches correspond to colors on the inset maps and Fig. 3

We analyzed 368 haplogroup Q3 samples from a diverse set of genealogical projects (see Methods for details) and assigned each to one of the Q3 branches. Figure 4 shows geographic places of origin for these individuals, thus indicating the geographic distribution of the branches.

Figure 4 links the deepest Upper Paleolithic split (Q3e *vs* the rest of Q3) to West and South Asia: most Q3e samples (green) were found in Pakistan and India, although some were also found in Europe and North Asia (in Kadom Tatars to the west of the Volga river and in a single Evenk sample from South Siberia); these latter samples fall into the narrower Q3e-YP4500 sub-branch and might represent a later spread of Q3e, rather than its place of origin.

The following splits were once again geographically linked to South and West Asia: most of Q3b samples (shown in red in Fig. 4) came from West Asia and neighboring parts of South and Central Asia, although one sample was found in Portugal. The areas of Q3c and Q3d could not be identified reliably as these branches were found in single samples. These two samples came from Poland and Inner Mongolia [42], respectively, indicating the wide geographic distribution of Q3 branches.

In contrast to these narrowly distributed clades, their brother clade Q3a is represented by multiple samples found mainly in West Asians and Europeans (Fig. 3). The remarkable phylogenetic structure of the clade has a clear reflection in geography. The ternary split of this branch resulted in a West Asian branch Q3a7 and two branches found in both Asia and Europe (blue Q3a6 and Q3a1’5; Fig. 4). The latter branch in turn split into five sub-branches (Q3a1 to Q3a5), some of which were West Asian and others European (Fig. 4 - yellow, purple, and brown). One of these European branches, Q3a5, was found in Germans and Dutch (Fig. 4, yellow), while another was found in Ashkenazi Jews (Fig. 4, brown) and accounts for 5% of their paternal pool (Table 1). This is the branch which split much later than the main set of phylogenetic events within Q3a, and whose sub-branches expanded between the second and tenth centuries AD.

## Discussion

### Origin, dissemination, and expansion of the haplogroup Q3-L275

Haplogroup Q3-L275 results from the first known split within haplogroup Q, which occurred in the Paleolithic epoch: according to previous studies [15, 24], haplogroup Q split into the Q3-L275 and Q1’2-L472 branches around 35 ky ago. Thus the location of this split might help identify the homeland of haplogroup Q, from where it spread throughout Eurasia and the Americas. Our findings better support a West Asian or Central Asian homeland of Q3 than any other area: a higher frequency was found in West Asia and in neighboring Pakistan; and early branches were identified in West Asia, Central Asia and South Asia. Increasing the dataset of ancient DNA might in future identify additional early branches, helping to locate a possible homeland more precisely. The very few samples from present-day (Additional file 3: Table S2) or ancient [43] China do not contradict this hypothesis, as they came from the western provinces located in Central Asia or historically linked to this area. The single Portuguese sample likely reflects the origin of the carrier, rather than more general population history. Thus, Q3 was one of the Paleolithic West Eurasian haplogroups. Its West/Central Asian homeland proposed here is hypothetical, because present-day genetic patterns do not necessarily reflect ancient ones as these can be modified by the more recent demographic events. Though TMRCA times of haplogroup emergence often do not correspond to demographic events, we note that the initial haplogroup Q3 breakup (around 15 ky BP, Fig. 3) is consistent with demographic changes in the post-Last Glacial Maximum period.

The following phases of Q3 history – between 14 and 4 ky BP – occurred again in West and Central Asia. These older branches did not reach Europe with the Neolithic wave (Fig. 2a) but one of the later branches – Q3a – is now found in both Asia and Europe. The fractionation of this branch around 4 ky BP allows us to speculate that it probably entered Europe from the Near East via historical contacts between states in Iran and the eastern Mediterranean, or from Central Asia via the North Pontic steppe along the chain of nomadic populations [14, 44, 45].

In Europe there are at least two branches: one in Dutch and Germans, and another in Ashkenazi Jews. These branches split from a common root 3000+/-700 years ago (Table 2, Additional file 2: Table S1): before the Jewish migration into Europe in Roman times [46]. Further screening in both Europe and the Levant is needed to determine whether the ancestors of the Ashkenazi acquired this lineage from the Levantine homeland or from the European host populations.

The aforementioned set of events belongs to the history of the Q3 lineage rather than the history of any specific population. We caution against interpreting branching patterns as simple signals of a bottleneck followed by a demographic expansion, because modern phylogenies represent the occurrence of the last bottleneck, not necessarily the only bottleneck the lineages have ever experienced.

The ages of the entire Ashkenazi cluster Q3a1 (1600+/-300 years ago) and its sub-clusters (1170+/-170 years ago; 1500+/-400 years ago, Additional file 2: Table S1) fall into the Early Middle Ages. Most scholars believe that at that time Ashkenazi ancestors lived in Europe, though they did not appear in written records with this name. The clear phylogenetic expansion within Q3a1 (Fig. 3), the expansion date, and nearby absence of Q3a1 in non-Ashkenazi Europeans indicate that this pre-Ashkenazi population – at least those who carried Q3 – grew rapidly and was already isolated in the Early Middle Ages.

### Lessons from collaboration of academic and citizen researchers

Our study was initiated by a citizen researcher [47] carrying out his research in association and with the support of the Genome Geography lab (Vavilov Institute for General Genetics, Russia), so the first lesson is that such collaborations can shed light on the phylogeography of rare haplogroups which might otherwise remain undescribed. Indeed, there were previously four sequenced Q3 Y-chromosome in the academic literature [24], while our study presents 47 more. This now makes Q3 one of the most extensively studied haplogroups in terms of the number of full Y-sequences relative to haplogroup carriers.

At least three branches of academic science – population genetics, forensic genetics and evolutionary genetics – provide extensive studies of Y-chromosomal variation in human populations. In the sphere of citizen science, Y-chromosomal variation is covered by the field called genetic genealogy, though it attracts attention of other citizen researchers as well. Genetic genealogical communities include many enthusiasts concentrating on one haplogroup each, while population geneticists (and other academic researchers) are normally involved in multiple projects and simultaneously analyze many haplogroups. So, genetic genealogists may offer specialization (elucidating phylogeographic details), while population geneticists might contribute generalization (extracting the principal patterns, and placing them into the overall picture of global genetic variation in humans).

Another difference stimulating mutual interest is the size of Y-chromosome sequence databases and strategies used to populate them. The genealogical databases contain thousands of sequenced Y-chromosomes [39, 48], which is larger by an order of magnitude than academic datasets (e.g. 456 Y chromosomes in [24]; 448 in [25]; 1244 Y chromosomes in [15]). However, genealogical databases contain many samples of unknown or doubtful population origin and these databases are drastically biased towards European-ancestry individuals. In contrast, Y chromosomes sequenced in academic research were often preselected to achieve uniform geographic or phylogenetic coverage and their population of origin is well-defined. Thus, one may consider whether the samples in academic databases are by an order of magnitude more valuable for phylogeographic analysis.

Apart from the full Y-chromosome sequences (the highest level of phylogenetic resolution), there are large databases of Y-chromosome genotypes with low to medium resolution. Both academic and citizen databases contain haplogroup frequencies and STR haplotypes in population samples, and in both cases the sample sizes vary from dozens to hundreds of individuals per population. There are two types of academic databases: the samples in population-genetic databases were collected from indigenous individuals, while the samples in forensic databases were collected from all individuals residing in the given area, without subdividing between individuals from indigenous groups and descendants of recent immigrants [49]. The samples in citizen science (genealogical) databases come from the customers themselves, and therefore are more similar to forensic databases, as they reflect the population of a customer’s country, including recent immigrants and temporary visitors. This is a problem for applying genealogical databases to historical and prehistorical reconstructions. Some genealogical projects (e.g. on the FamilyTreeDNA website) try to solve this problem by organizing the customer samples according to their deepest known paternal origin rather than by country of present-day residence. There are genealogical projects for many countries and ethnic groups. The same country or populations were often studied in academic research, allowing direct comparison of the results (Table 1). The higher Q3 frequencies from both datasets are in general agreement. For example, the frequency of Q3 in Ashkenazi ranges from 1% to 5% according to academic, and from 2% to 6% according to genealogical, datasets; in Pakistan and India it ranges from 0% to 5% according to academic data and from 1% to 9% according to genealogical datasets (Table 1). In populations where Q3 is rare, both similarities and differences of two datasets could be stochastic. Thus, to compare the datasets we looked at their overall patterns revealed by the gene geographic maps (Fig. 2a *vs* b). Q3 is present on both maps in West and South Asia and absent or very rare in other parts of Asia. But the maps differ in their European sections: academic data indicate very few spots and very low frequencies (Ashkenazi data are not shown), while genealogical projects show from 0% to 5% frequency of Q3 (again, data from Jewish projects are not shown). This difference could be caused by two factors. First, there are some rare Q3 branches which are present in both indigenous and general populations, but occasionally were missed in academic samples (like Q3e in Tatars). Second, individuals of paternal Jewish ancestry might appear in non-Jewish genealogical projects, but were not included in academic samples due to strict sampling criteria and genealogical information collected during sampling.

Both academic and citizen scientists of our team believe that, despite genealogical projects often providing important data, overestimating their information value should be avoided. For example, the differences in haplogroup frequencies from academic data on the same population could be caused by peculiarities of genealogical sampling. The social activity and ethnic origin of a project’s administrators and participants can make a genealogical sample non-random, as it tends to include clusters of relatives (or persons belonging to the same genealogy according to historical records or oral tradition), or is biased to a particular geographic locality. Genealogical projects differ in the criteria they apply for including customer samples. Thus projects which provide results similar to academic data on the same population reliably describe the gene pool of stable regional/ethnic populations. Data from these projects can then be used along with academic datasets, increasing the total sample size and revealing additional genetic lineages. It should be noted, however, that as mentioned geographic coverage in genealogical projects is strongly biased to Europe, and there are many non-sampled regions in Asia (Fig. 2b), in contrast to the larger number of populations and global coverage achieved in academic datasets (Fig. 2a). This difference should also be considered when pooling both kinds of data.

Finally, geneticists and genealogists often differ in their approach to interpreting the data. Geneticists typically consider the entire gene pool, consisting of many haplogroups, and draw conclusions based on their relative frequencies in different populations. Even in studies dedicated to a single haplogroup, conclusions about population history take into account the data from other components of the gene pool. Genealogists, on the contrary, focus on the history of a single lineage. This is fully justified for understanding the genealogy of an individual, as he has a single paternal lineage. But there is no ‘genealogy of populations’: a population does not have a single root (linear history), marked by a specific haplogroup. Instead, a population typically consists of individuals with different origins and different haplogroups. As a result, the “one haplogroup history – one population/ethnic history” hypothesis would be a dangerous oversimplification, and in addition inappropriately expands the history of an ethnic group to a time depth far exceeding the formation the group itself (its identity and language). We believe that haplogroups indeed mark features of population history, but caution should be observed when combining these features into an entire picture. Different approaches should be used for reconstructing individual (one lineage) and population (multi-lineage) paternal histories, and collaborative studies of geneticists and genealogists can help to avoid one-sided interpretations.

## Conclusions

Haplogroup Q3-L275 represents the oldest known split within Eurasian/American haplogroup Q, and most likely occurred in West or Central Asia in the Upper Paleolithic period. It could be hypothesized that during the Mesolithic and Neolithic epochs, Q3 remained a minor component of the West Asian Y-chromosome pool. It gave rise to five branches (Q3a to Q3e) which spread across West, Central and parts of South Asia. Around 3–4 millennia ago (Bronze Age), the Q3a branch, like several other Y lineages [15], underwent a rapid expansion, splitting into seven branches, some of which entered Europe. One of these branches, Q3a1, was acquired by a population ancestral to Ashkenazi Jews and grew within this population during the 1st millennium AD, reaching up to 6% in present-day Ashkenazi.

This study dataset was generated by a Y-chromosome genotyping effort in the genetic genealogy community, and phylogeographic patterns were revealed by a collaboration of population geneticists and genetic genealogists. This positive experience of collaboration between academic and citizen science provides a model for further joint projects. Merging data and skills of academic and citizen scientists promises to combine, respectively, quality and quantity, generalization and specialization, and achieve a well-balanced and careful interpretation of the paternal-side history of human populations.

## Methods

### Study design

The data used in this study came from three sources: academic papers on Y-chromosome variation in human populations, genealogical projects, and genome variation databases.

Frequencies of haplogroup Q-M242 were obtained from published academic papers and plotted on a map (Fig. 1a).

Frequencies of haplogroup Q3 were obtained from both academic papers and citizen genealogical projects and plotted independently on two maps: Fig. 2a resulted from academic data, while Fig. 2b and Additional file 1: Figure S1 resulted from genealogical projects data; the only difference between the last two maps is the absence or presence, respectively, of the data from Ashkenazi projects.

For 47 Q3 individuals, we obtained full Y-chromosome sequences (Additional file 4: Data S1). These sequences were used to create a phylogenetic tree (Fig. 3), identify branches (Additional file 2: Table S1) and estimate their ages (Table 2).

By using and updating all three aforementioned sources, we identified 354 haplogroup Q3 carriers for whom the branch within haplogroup Q3 was known (Additional file 3: Table S2). Most of these individuals were plotted on a map according to their place of paternal origin, thus showing the geographic distribution of each branch (Fig. 4).

### Analysis at the phylogenetic level of haplogroup Q

Frequencies of haplogroup Q-M242 were extracted from our in-house *Y-base* database [50], containing data on 144,464 Y-chromosomes (3,670 population samples) collected from 238 papers (references not shown). Samples whose geographic origin was not precisely indicated in the source paper, or which were not indigenous to the place of sampling, were excluded; small population samples were either excluded or pooled with geographically close samples of the same ethnic group. This resulted in a dataset of 126,155 Y-chromosomes from 1000 populations. Among these, 480 population samples (total sample size 65,528) were either directly typed for M242, or the absence of M242-derived chromosomes was clear because 100% of Y-chromosomes fall in other haplogroups. This dataset from 480 world populations was used to construct the frequency distribution map of haplogroup Q-M242 (Fig. 1a) by the GeneGeo software [51, 52] with weight function set to 3 and radius of influence set to 1,500 km.

### Analysis at the phylogenetic level of haplogroup Q3

To reveal the distribution of haplogroup Q3-L275, we used both published data from the academic literature, and data from genetic genealogical projects.

The academic data on frequencies of haplogroup Q3-L275 in indigenous populations were extracted from published papers (Table 1). Published STR-profiles and the Y-chromosomal haplogroup predictor [53] were additionally used to identify likely Q3 samples among haplogroup Q-M242 sets. The prediction rules are listed in the Additional file 5: Table S3.

The citizen science data from haplogroup Q3-L275 were collected across all genealogical projects represented on the FamilyTreeDNA website. Where samples were not typed for L275 or M378, their STR profiles were used to predict their status. Predictions were performed with Family Tree DNA’s prediction algorithm and by the YPredictor [53], following the prediction rules (Additional file 5: Table S3).

Table 1 presents all populations or projects where we identified non-zero frequencies of Q3-L275. The samples which were predicted to belong to Q3 are marked by an asterisk in the Table 1; all other samples were directly confirmed by SNP-testing.

Both academic data on Q3 presence in indigenous populations (Table 1, left column) and citizen science data on Q3 presence among genealogical customers from different countries or ethnic groups were independently plotted on the two maps. As Jews are not typically considered as indigenous Europeans, the data on Ashkenazi Jews were not used for the map based on academic data (Fig. 2a). Thus, the map based on genealogical data was constructed in two forms: Additional file 1: Figure S1 includes, and Fig. 2b does not include, data on Ashkenazi Jews. The maps were constructed by the GeneGeo software as described above.

### Full Y-chromosome sequencing

We have created a dataset including 47 sequenced Y-chromosomes of haplogroup Q3. Most of them (40 samples) were sequenced within the framework of the Y-DNA Haplogroup Q-M242 genealogical project [54] and are presented here for the first time (Additional file 6: Table S4). Screening of genome variation databases [55–58] identified seven samples representing haplogroup Q3 [15, 29, 58], which were also included in our analysis (Additional file 6: Table S4).

30 out of 47 samples were sequenced by the BigY technology commercially available at Gene by Gene, Ltd [48] and covering 11 Mb of the Y-chromosome. The remaining 17 samples were sequenced by five different approaches (see Additional file 6: Table S4 for details).

Twenty four sequenced Y-chromosomes came from European populations, ten from West Asia, five from South Asia, three from Central Asia, three from the Caucasus, one from East Asia and one from North Africa (Additional file 6: Table S4).

### Phylogenetic analysis

We analyzed a dataset of 48 high-coverage Y-chromosome sequences, including 47 samples representing haplogroup Q3 and one haplogroup Q1 sample, Kazakh-2-Q1a (used as outgroup). This dataset was heterogeneous, as it was produced by different hardware platforms and software toolkits. Almost all data were available in BAM file format, except for the three samples obtained in CGATools masterVar files. To produce a phylogenetic tree of sufficient quality and resolution, we used the following pipeline.

BAM files were pileupped using Samtools ver. 1.2 [59] and the output was processed with NGSConv (this in-house software developed by the authors was used here to apply filtering rules to the raw VCF and to format contigs for the phylogenetic software). All indel positions were realigned using the code from the Seqan [60] library and then excluded. Generally, we kept SNP positions near indels, except for the regions with high probability of containing STRs (for this, we measured the “dimeric” entropy value calculated in a the way close to that of lobSTR [61]). Rules in the general form (those operating with variables such as ‘read count’ or ‘base/mapping quality’) were always preferred over hard-coded exclusion of a particular position or a range. To prevent the loss of phylogenetic resolution due to a limited overlap of the sequencing ranges in different samples, we started with extremely relaxed rules for SNP filtering. We subsequently tightened the rules for calling and filtering, each time constructed a number of intermediate phylogenetic trees using the Phylomurka software [62], and evaluated their quality by phylogenetic criteria: mean Robinson-Foulds (RF) distances between the optimal MP trees and the number of characters with parallel or recurring changes. For example, we found that such rules as base quality values less than 15, entropy threshold for excluding STR regions greater than 0.44 and call rates below 0.6 still resulted in trees of reasonable topologies, but the mean RF distances between them exceeded 0.15, which we considered evidence of errors in the data or substantial phylogenetic uncertainty. Finally, this iterative process converged to a unique MP tree (Additional file 2: Table S1) with the following rules for data selection: read depth > = 2; base quality > = 15 and mapping quality > = 10; entropy threshold > = 0.44; “heterozygous” positions (i.e., if different reads indicated more than one allele) were excluded only if the minor allele frequency exceeds 10%; the call rate for almost all positions was > =0.6 (i.e. a position is taken into account if it is covered in at least 60% of samples), but a lower call rate was accepted for several SNPs used to name the branches. The final alignment contained 1614 variable positions in a 9.34 Mb region of the Y-chromosome (see Additional file 7: Table S5). The data retrieval and phylogenetic features of the final alignment are summarized in Additional file 8: Table S6. The robustness of our final solution was then tested by parsimony bootstrapping using the TNT software [63] and by ML bootstrapping with the PhyML [64] and RAxML software [65]. These tests showed almost the same topology, with only three branches (each defined by a single SNP) not passing the bootstrap threshold of 50%, although most other tree partitions were supported by more than 90% of replicas.

Selection of the read depth and call rate thresholds deserve additional comments. The minimum read depth settings and the threshold for accepting “false heterozygotes” (for the haploid human Y chromosome) are essentially ambivalent in their effect on the overall process. More “stringent” settings usually increased the level of phylogenetic uncertainty. Conversely, allowing a small proportion of “minor” alleles (say, when 10% alleles are different from one finally deduced) provided more support for particular clades which otherwise were left unresolved. In our sample, both “stringent” (read depth at least 10 and only one allele) and relaxed (read depth at least 2 and at most 10% of the minor allele) settings led to the generally similar topologies and same level of phylogenetic “noise” (4–5 recurring or parallel changes) but the tree constructed under the “relaxed” criteria (Additional file 9: Figure S2) had better resolution compared to one obtained with the “stringent” settings (Additional file 10: Figure S3). When selecting the value of the call rate, we considered the following conditions: (1) The ability of the software to infer the values of missing nucleotide states as uniquely as possible, without expanding the optimal solution space to an impractical size; (2) The degree to which the sequenced ranges overlap: more than a third of our sequences are 1.5–3.5 times longer than the remaining ones; (3) The variation of root-leaf distances (in nucleotide changes) in the resulting trees. We found that the phylogenetically-motivated value of 60% for the call rate still makes the size of the common region (9.34 Mb approximately) close to the average size of our “shorter” samples (9.1 Mb). We actually have a negligible variation of the root-leaf change numbers in our best tree: the corresponding value is 118 + -8; thus our call rate value does not distort the tree metric and the contribution of “private” mutations in samples with long covered ranges is very limited. The main outcome of these bioinformatic experiments is not the superiority of a particular way to deal with heterogeneous NGS data, but rather a demonstration that a set of quite simple rules may lead to very consistent phylogenetic results.

To estimate the divergence times of the entire haplogroup Q3 and its sub-branches, we applied the rho-statistic (as described in [66]). To avoid platform and coverage bias, we only used a subset of 30 samples sequenced by the BigY technology (these sample names are shown in italic in Fig. 3 and Additional file 2: Table S1). Note that restricting to samples sequenced by the same platform does not affect the topology of the tree (Additional file 11: Table S7) though, of course, it leads to missing the branches represented by the samples sequenced by other approaches. We used the calibration from [26], obtained by sequencing the Y-chromosomes of nine individuals with a deep common genealogy. Hardware and software conditions of sample processing from [26] were nearly identical to those we have applied to the 30 BigY samples in this study; in particular, these conditions include higher call rate and stronger filtering rules than those used for the whole dataset of 48 samples. The rate of the Y-chromosome SNP mutations calculated in [26] as 0.78 × 10-9 per bp per year yields the value of Q3 TMRCA as 15,100 + -1200 years.

To name the haplogroups, we followed the notation suggested in [24]. Table 2 and Additional file 2: Table S1 provide the alternative names and defining markers for haplogroups mentioned in the text.

An independent phylogenetic analysis of roughly the same dataset of sequenced Y-chromosomes was performed manually by a group of citizen scientists, coordinated by the administrators of the Haplogroup Q genealogical project [54]. The resulting tree is presented in Additional file 12: Table S8, while Additional file 13: Table S9 contains the additional SNPs identified. One may see that this “citizen science tree” (Additional file 12: Table S8), although differing in format from our main tree (Additional file 2: Table S1), reveals almost the same topology.

### Analysis at the phylogenetic level within haplogroup Q3

To subdivide Q3 samples according to their subclades, we identified 414 haplogroup Q individuals (45 from academic papers and 369 genealogical customers). 46 out of the 414 were of unknown origin (a person has provided his genotype but has not provided any information about paternal origin); these samples were not used for analysis, thus decreasing the total sample size to 368 (details of these samples are presented in Additional file 3: Table S2). Among them, 132 samples were typed up to major trunks only (L275, M378, L245) and thus were not suitable for finer phylogenetic analysis. Also, five samples representing three very rare branches were not considered (Additional file 3: Table S2). However, the remaining 231 samples were typed phylogenetically deeply, and their place of paternal origin was known, thus allowing cartographic analysis of their distribution. As the number of samples was too low to draw frequency distribution maps, we just mapped the place of paternal origin for every sample (Fig. 4).

Among the 231 samples used for the mapping, most were genotyped at branch-defining SNPs in the framework of the haplogroup Q genealogical project, mainly under supervision of members of this study research team. Also, we genotyped the Q3 Marsh Arab samples from [30] for downstream markers and used the samples with full Y-chromosome sequencing. For 188 out of 231 samples, their branch within Q3 was directly tested at the corresponding SNP marker. The remaining 43 samples were predicted from their STR-profiles, as Additional file 3: Table S2 indicates. Among them, 40 samples were typed by 67 (or 111) STR-markers and were assigned to the branch if they demonstrated less than 6 mutation steps from a sample(s) which was assigned to this branch by a SNP. (31 of them were predicted to carry the Ashkenazi-specific Q3a1-Y2200 branch and were of Ashkenazi origin, which additionally confirms the reliability of our prediction). The remaining three samples were typed by 12 or 37 STR markers and were predicted to carry the Q3e-Y1150 branch, typical of Kadom Tatars, because they have zero or one mutational steps from samples assigned to this branch by a SNP, and have Tatar origin.

## Additional files

Additional file 1: Figure S1. Frequency distribution map of haplogroup Q3-L275 in total contemporary populations (citizen science databases including Ashkenazi Jews projects, total sample size 27,922). (JPG 4519 kb)
Additional file 2: Table S1. Detailed phylogenetic tree of the haplogroup Q3-L275. 47 Q3-L275 samples and 1 Q1a-M346 outgroup. Constructed with the Phylomurka software using MP criteria, from the alignment obtained with read depth > = 2, base quality > = 15 and mapping quality > = 10, call rate = 60%. (XLSX 279 kb)
Additional file 3: Table S2. Genotypes of the individual samples. (XLSX 36 kb)
Additional file 4: Data S1. VCF and BED files for the sequenced Y-chromosomes. (RAR 12315 kb)
Additional file 5: Table S3. The rules for predicting haplogroup Q3-L275 from STR profiles. (XLSX 10 kb)
Additional file 6: Table S4. The full Y-chromosomal sequences of haplogroup Q3-L275. (XLSX 11 kb)
Additional file 7: Table S5. The dataset of Y-chromosome SNPs observed in the haplogroup Q3-L275 samples sequenced in this study. (XLSX 365 kb)
Additional file 8: Table S6. Data retrieval and phylogenetic features of the sequence alignment used for the tree. (XLSX 14 kb)
Additional file 9: Figure S2. Phylogenetic tree of haplogroup Q3-L275 with read depth value ranged from 2. 44 Q3-L275 samples and 1 Q1a-M346 outgroup. Constructed using ML GTRGAMMA model with the RAxML software, from the alignment obtained with read depth > = 2, base quality > = 15 and mapping quality > = 10, call rate = 60%. Three samples sequenced with the Complete Genomic (CG) technology were excluded due to problems with estimating their exact read depth values. The topology is the same as shown in the main tree (see Additional file 3: Table S2) except for the three omitted GC samples, although the method of construction is different. (JPG 79 kb)
Additional file 10: Figure S3. Phylogenetic tree of haplogroup Q3-L275 with read depth value ranged from 10. 44 Q3-L275 samples and 1 Q1a-M346 outgroup. Constructed using ML GTRGAMMA model with the RAxML software, from the alignment obtained with read depth > = 10, base quality > = 15 and mapping quality > = 10, call rate = 60%. Three samples sequenced with the Complete Genomic technology were excluded due to problems with estimating their exact read depth values. The topology is less refined compared to that obtained with read depth value > = 2 (see tree 2). (JPG 76 kb)
Additional file 11: Table S7. Phylogenetic tree of the haplogroup Q3-L275 based on samples sequenced by one particular technology (Big Y). (XLSX 119 kb)
Additional file 12: Table S8. Phylogenetic tree of the haplogroup Q3-L275 developed by citizen scientists (administrators of the Y-DNA Haplogroup Q-M242 Project [54]). (XLSX 39 kb)
Additional file 13: Table S9. The additional set of Y-chromosomal SNPs polymorphic in the haplogroup Q3-L275 samples identified by citizen scientist (administrators of the Y-DNA Haplogroup Q-M242 Project [54]). (XLSX 42 kb)
