# Supplementary figures and images for: Phylogeography of human Y-chromosome haplogroup Q3-L275 from an academic/citizen science collaboration
Source: BMC Evol Biol. 2017 Feb 7;17(Suppl 1):18. doi: 10.1186/s12862-016-0870-2 (PMC5333174; doi:10.1186/s12862-016-0870-2)

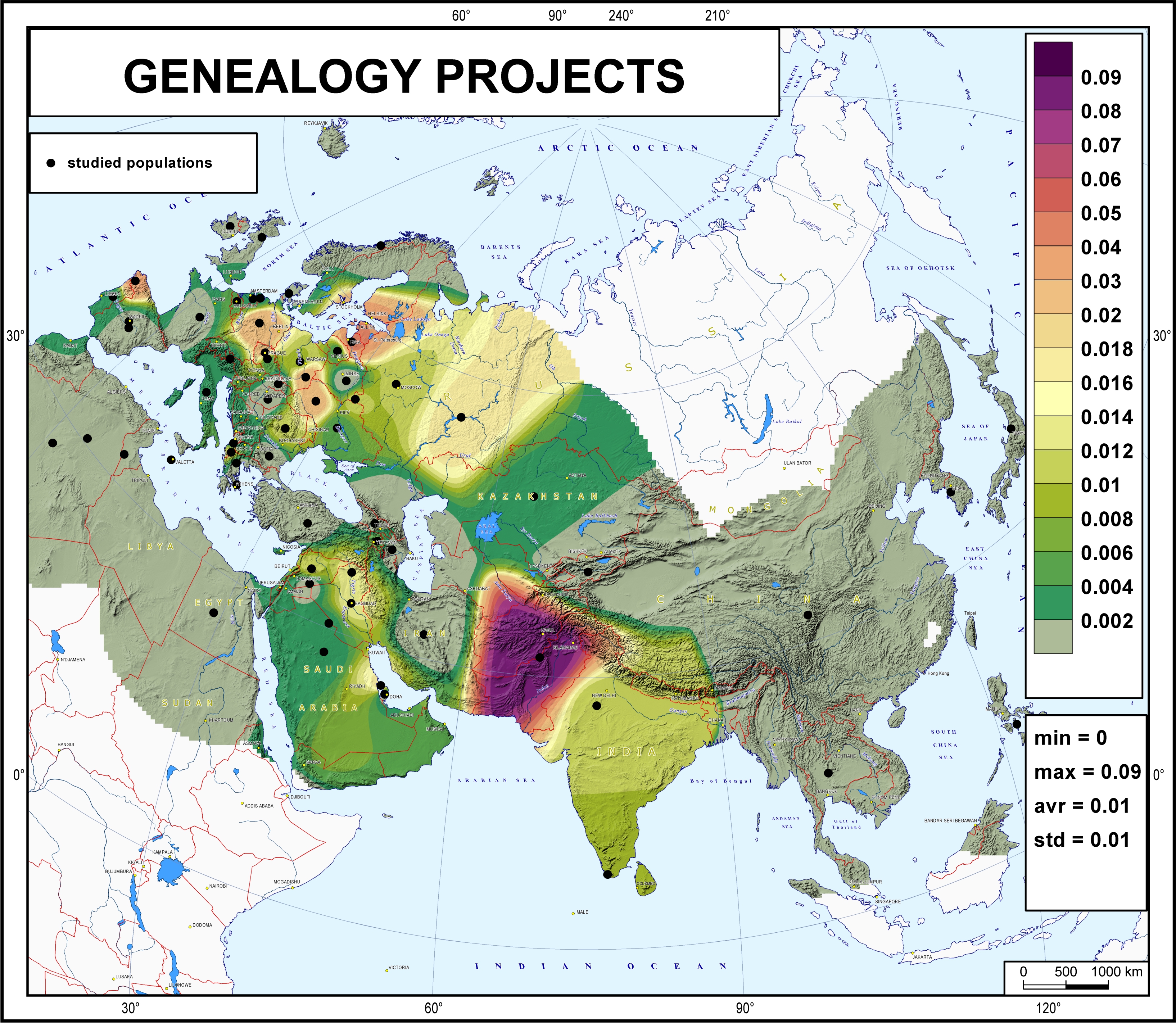

Supplement: Additional file 1: Figure S1. — Frequency distribution map of haplogroup Q3-L275 in total contemporary populations (citizen science databases including Ashkenazi Jews projects, total sample size 27,922). (JPG 4519 kb) [file 12862_2016_870_MOESM1_ESM.jpg]

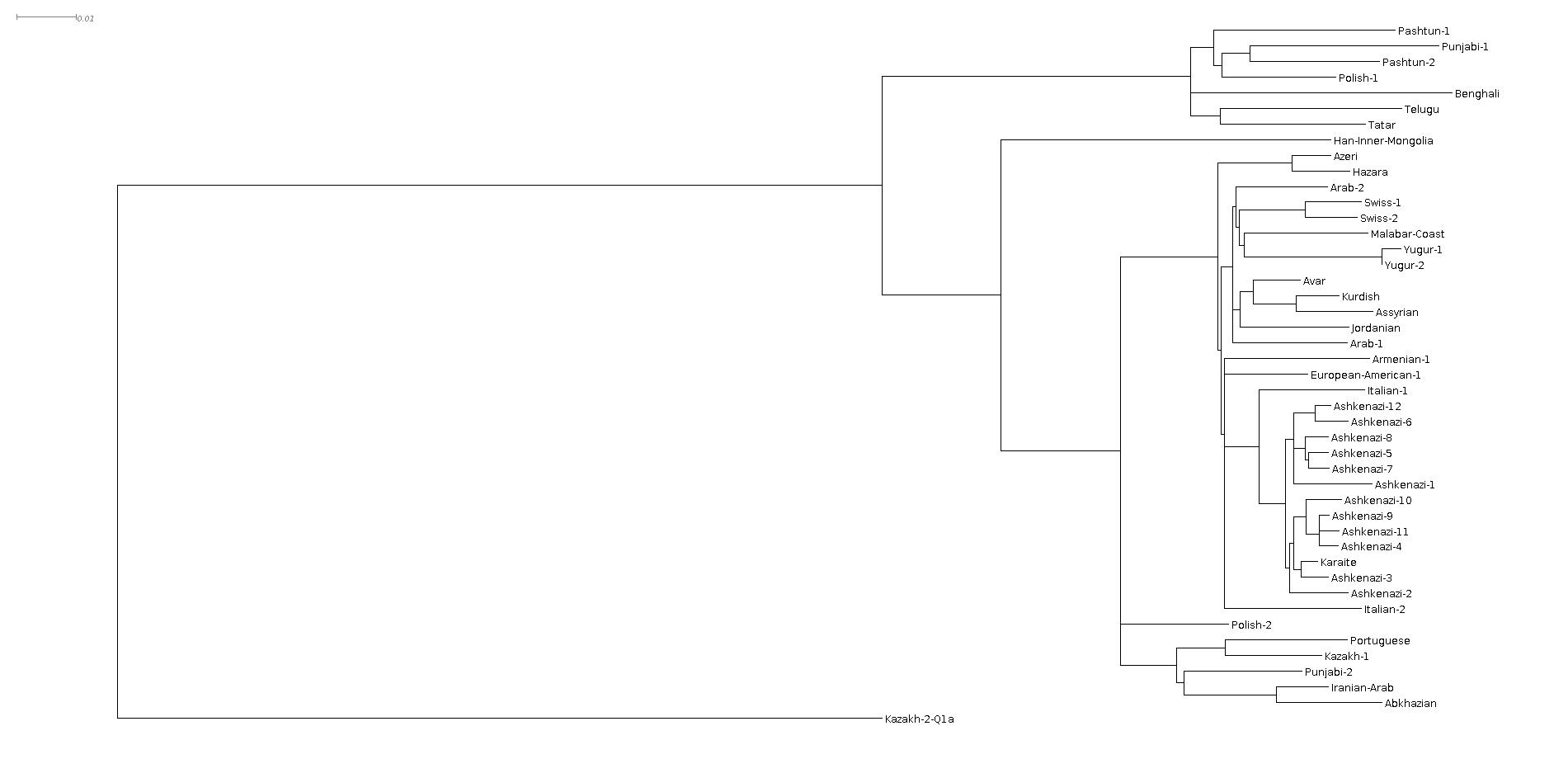

Supplement: Additional file 9: Figure S2. — Phylogenetic tree of haplogroup Q3-L275 with read depth value ranged from 2. 44 Q3-L275 samples and 1 Q1a-M346 outgroup. Constructed using ML GTRGAMMA model with the RAxML software, from the alignment obtained with read depth > = 2, base quality > = 15 and mapping quality > = 10, call rate = 60%. Three samples sequenced with the Complete Genomic (CG) technology were excluded due to problems with estimating their exact read depth values. The topology is the same as shown in the main tree (see Additional file 3: Table S2) except for the three omitted GC samples, although the method of construction is different. (JPG 79 kb) [file 12862_2016_870_MOESM9_ESM.jpg]

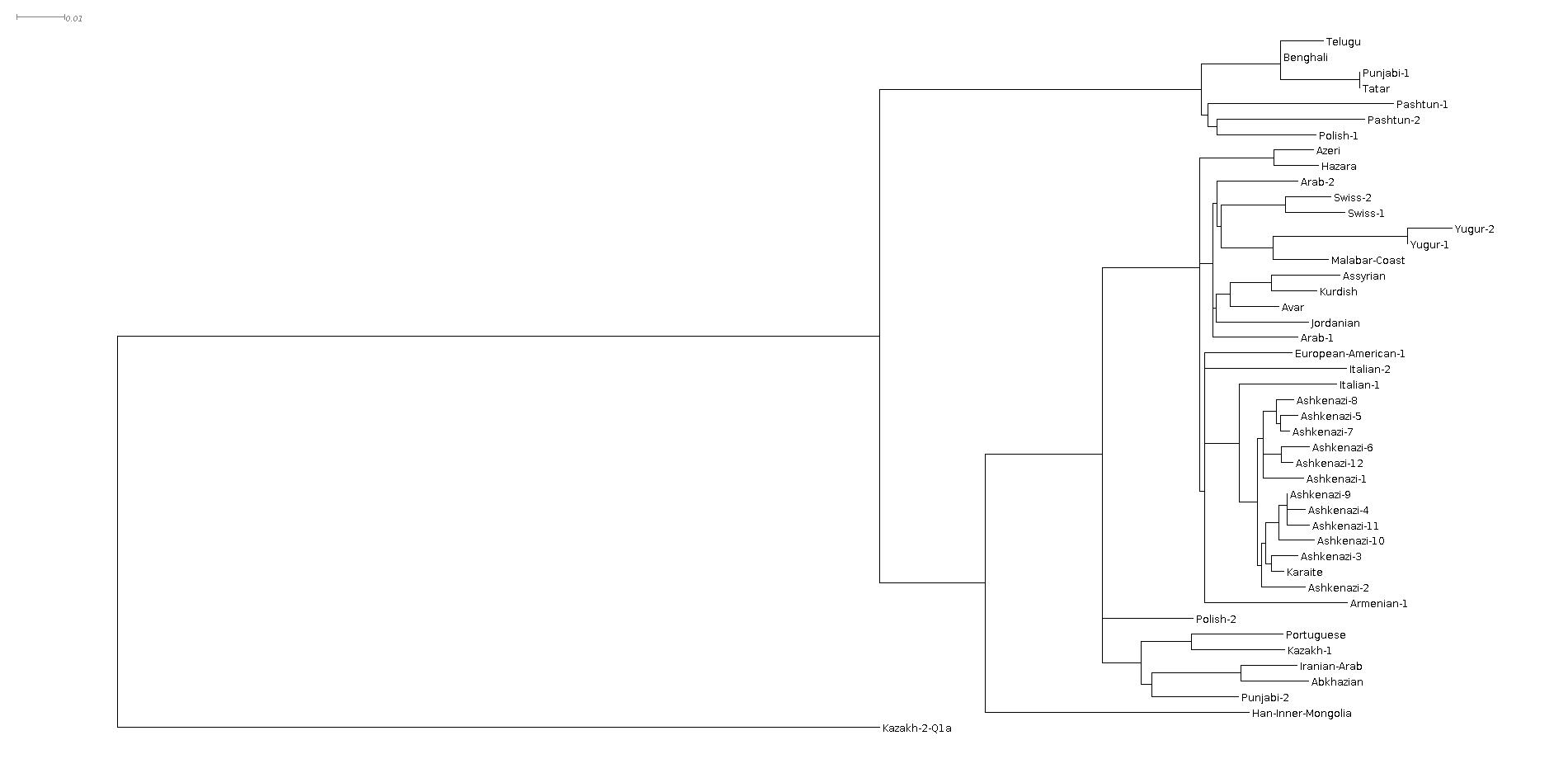

Supplement: Additional file 10: Figure S3. — Phylogenetic tree of haplogroup Q3-L275 with read depth value ranged from 10. 44 Q3-L275 samples and 1 Q1a-M346 outgroup. Constructed using ML GTRGAMMA model with the RAxML software, from the alignment obtained with read depth > = 10, base quality > = 15 and mapping quality > = 10, call rate = 60%. Three samples sequenced with the Complete Genomic technology were excluded due to problems with estimating their exact read depth values. The topology is less refined compared to that obtained with read depth value > = 2 (see tree 2). (JPG 76 kb) [file 12862_2016_870_MOESM10_ESM.jpg]
